# Lessons learned from applying a “Rapid Maternal Death Surveillance and Response” tool in conflict-affected Tigray, Ethiopia

**DOI:** 10.1186/s12884-025-08182-y

**Published:** 2025-10-06

**Authors:** Benjamin Black, Noortje Rijnberg, Kibrom Solomon, Desta Kahsay, Mehari Gebru, Berhane Fseha Teklehaimanot, Solomon Hintsa, Caren Asilwa, Maura Daly, Crystal Van Leeuwen, Ruth Kauffman, Hanna Majanen, Anne Marie Morales, Alan De Lima Pereira, Monique Pereboom, Hale Teka

**Affiliations:** 1 Médecins Sans Frontières Operational Centre of Amsterdam, Plantage Middenlaan 14, Amsterdam, 10188 DD The Netherlands; 2 Suhul Government Hospital, Shire Town, Tigray Region Ethiopia; 3 Head School of Public Health Sciences and Comprehensive Specialized Hospital, Aksum University, Aksum, Tigray Region Ethiopia; 4 Department of Obstetrics and Gynaecology, College of Health Sciences, Mekelle, Tigray Region Ethiopia

**Keywords:** MPDSR, Humanitarian, Maternal health, Quality improvement, Tigray, MISP, Anaemia, Maternal mortality

## Abstract

Women in humanitarian settings face high maternal mortality, yet Maternal Death Surveillance and Response (MDSR) systems are lacking. Medical teams in Tigray, Ethiopia, designed and piloted a *rapid* MDSR tool. The tool identified actions to improve maternity care within the humanitarian response. Adapted MDSR tools may be beneficial in humanitarian crises however further research is required.

**Supplementary Information:**

The online version contains supplementary material available at 10.1186/s12884-025-08182-y.

## Introduction

Ethiopia has made significant progress in reducing its maternal mortality ratio (MMR), demonstrating a 72% reduction between 2000 and 2020 [1]. Nonetheless, it remains one of four countries in the world reporting over 10,000 maternal deaths per year [1]. Tigray region, in Northern Ethiopia, also witnessed a significant decrease in the MMR, declining from 266 to 186 per 100,000 live births in the period between 2013 and 2019 [2, 3].

In November 2020, a conflict in Tigray region of Ethiopia, resulted in the displacement of over two million people and a humanitarian crisis of staggering proportions [4, 5]. The conflict prevented aid or international observers from entering the affected areas [6]. Following widespread destruction and looting of health facilities, only 3.6% were left functional [7]. According to the United Nations Populations Fund (UNFPA), 83% of facilities previously able to provide services for pregnant women were no longer capable [8]. The Tigrayan MMR was estimated to have risen over fivefold from 186 pre-conflict to 840 deaths per 100,000 livebirths [9].

Before the conflict, the Tigray region was implementing maternal and perinatal death surveillance and response (MPDSR). A 2019 study found 54.5% of Tigrayan health facilities were practising good quality death reviews and 53% taking actions as part of MPDSR [10]. However, the conflict interrupted MPDSR in many facilities across the region due to loss of staff and overwhelmed health systems.

Médecins Sans Frontières Operational Centre of Amsterdam (MSF-OCA) was one of a few humanitarian aid agencies to respond in scale to the medical humanitarian crisis in Tigray. In June, 2021, outreach teams noticed in their records that babies were being brought to clinics without their mothers in the area around Sheraro town. In the first 11 weeks of MSF’s intervention in this area, 13 babies were presented without living mothers [11]. There were further reports of women dying in childbirth at home, being unable to reach a functioning healthcare facility along with limited community knowledge for safe homebirth [6]. Due to the emerging information that these maternal deaths were taking place, more investigation was necessary. To respond, MSF-OCA worked with local stakeholders, (including the hospital director, obstetricians and midwives), at Suhul Hospital in Shire Town, the only operational health facility providing comprehensive emergency obstetric and newborn care (CEmONC) across a large swathe Tigray for local and displaced populations [12, 13], to develop and implement a maternal death surveillance and response (MDSR) tool adapted to the humanitarian emergency setting.

This Comment reflects on our experience and lessons learned from the development, design and pilot testing of this MDSR tool during the conflict and linked humanitarian crisis in Tigray between 2021–2023.

## The need for MPDSR implementation in humanitarian crises

According to UNFPA, approximately two thirds of all maternal deaths occur in countries affected by “humanitarian crisis or fragile conditions [14]”. This increased risk equates to a maternal mortality ratio in humanitarian settings which is nearly double the global estimate [6]. Despite this, the application of MPDSR in humanitarian settings prior to the conflict in Tigray had been inconsistent and problematic [15]. Many of the challenges documented echo similar descriptions from low resource countries, such as overburdened services, lack of training and blame culture; however, these are intensified by additional political, economic, social and security constraints typical of crises [16, 17]. Implementation guidance for MPDSR was released by WHO in 2021 and includes a chapter dedicated to humanitarian settings, recognising some of these unique challenges [18]. The guidance advises that MPDSR should not be implemented in the acute phase of a crisis but can be considered once the situation has stabilised.

While there’s an acceptance of increased risks to maternal health during humanitarian crises, there is often no system to identify *and respond* to the causes [19]. Without this process, there is a risk of general public health responses, which may overlook key context-specific challenges, allowing assumptions to lead actions rather than a nuanced root-case analysis approach. Although MSF-OCA had several years of experience implementing MPDSR in stabilised humanitarian settings, it had not achieved this during an emergency response. The situation in Tigray demonstrated the need for a framework to recognise, review and prevent further maternal deaths in the humanitarian emergency setting, as the system established prior to the conflict was no longer functioning. Thus, MSF-OCA set out to design and pilot test a simplified MDSR tool in Tigray.

## Development and design of the tool

The development of the MDSR tool was a collaborative process merging the expertise of reproductive health and humanitarian emergency teams. The tool was developed through an iterative process that involved discussions with Ministry of Health colleagues and locally recruited health care staff who were on the frontlines of providing care, aid workers, maternal health specialists, and the MSF-OCA Emergency Support Desk (ESD), the department which responds to acute humanitarian crises. End-users of the tool, notable frontline health workers and managers and aid workers, were engaged throughout the development and implementation processes as they understood the context of the emergency best. The discussions of how to adapt MDSR for the humanitarian emergency setting reflected the challenges, and potential compromises to the process, for its inclusion alongside the many other competing priorities placed upon teams. The intention was to produce a tool which wouldn’t be arduous for busy teams, taking a pragmatic approach to the amount of detail required and reducing bureaucratic processes. This reduced version of MPDSR was named “rapid maternal death surveillance and response (R-MDSR)”.

The R-MDSR tool had three parts. Part A included key demographic and clinical aspects of the woman and her death enabling the team to be able to identify patterns in geographic spread, timeline and causes of deaths. Part B was for the identification of root causes and plan for action points using an *After Action Reviews* [20] approach. Part C provided space to record what had been learnt and what actions were planned for improving future outcomes. The information was completed on R-MDSR forms and sent to a focal person who would transcribe the information to the R-MDSR spreadsheet, with patient details anonymised, which was then reviewed on a regular bases by the facility team for monitoring and planning. For further description, and example, of the R-MDSR tool refer to additional files 1 and 2.

The priority was a tool which would be useful in the humanitarian emergency setting. The design team balanced more thorough approaches to MPDSR, as recommended in the WHO guidelines, against the risk of health workers not attempting reviews at all and adapted accordingly. For example, R-MDSR tool accepted limited or partial information since it was expected that there would be gaps in information due to the stressful and disorderly circumstances of an emergency.

Additionally, the stakeholders involved in the development of the tool prioritised maternal deaths, aiming for all deaths to be reviewed through the tool. Where capacity allowed, inclusion of near miss cases was also encouraged. Perinatal deaths were not included, to focus the teams on maternal health within this limited context. If teams were able to include perinatal deaths, they were encouraged to transition across to a standard MPDSR rather than continuing with the rapid tool.

Similarly, the tool allowed for recording action points for informing later activities when the situation had stabilised acknowledging that the teams completing the tool may not have the capacity during the emergency to immediately work on quality improvement. R-MDSR was designed with both facility and community deaths in-mind, recognising that in the turmoil of the early stages of an emergency, there may be significant overlap and confusion between the two.

## Pilot testing the tool

MSF-OCA piloted the R-MDSR tool in Suhul Hospital over a period of 8-weeks starting in January 2023 and then reviewed. The initial focus was on facility-based deaths, as logistical and security challenges hindered access to distant communities.

Eight maternal deaths were identified at Suhul Hospital, and the R-MDSR tool was utilised for all cases by the hospital team. The completeness of information varied; present healthcare workers were interviewed for all eight cases, but medical files were only found for five women. However, the R-MDSR tool allowed for these data gaps. None of these deaths had been entered into the hospital's maternal mortality registration or subjected to a formal maternal mortality audit due to the overwhelmed health system at the time.

Among the eight documented maternal deaths, five were attributed to severe anaemia, resulting in organ failure (non-haemorrhagic). Four of these occurred in the antenatal period and one was a delayed postpartum death. All cases reviewed, and actions identified, were recorded into an R-MDSR excel spreadsheet, with patient details anonymised. The spreadsheet served as a communication tool within the organisation and provided institutional memory for turnover of international and local staff. The updated version was shared by the maternity lead, who had overall ownership and control of the tool.

Using the tool, the hospital team was able to review these cases among a small group and identified that pregnant women were admitted to various medical wards among other general inpatients, with no specialised antepartum area or dedicated staff with maternity experience. Blood was almost impossible to receive due to conflict-affected dysfunction of the pre-war centralised blood transfusion service and supply gaps for enacting local crossmatch and screening.

The findings from the R-MDSR tool were presented to operational managers with an emphasis on addressing the action points identified. Since during the pilot most cases related to severe anaemia, the operational managers used the data from the tool to propose several strategic interventions:

1. A specialised clinical area for antepartum pregnant women.
2. Focused, qualified, skilled attendants to work in this area and advocate for their patients.
3. Support to the hospital laboratory and regional blood bank with transport, blood donation drives, and medical supply to increase availability of safe blood transfusions.

## Results from the pilot test

After three months of implementing these strategies, from February 2023 till April 2023, there were 92 antenatal admissions to the dedicated antenatal area, 71% of women had severe anaemia (haemoglobin below 7 g/dL) and 53% had a haemoglobin of below 4 g/dl. All women admitted remained in or near the hospital after treatment to await birth. There were no maternal deaths among these women.

The data collected through the review process, though limited in detail, focused the team’s attention on the main causes, and contributing factors, for maternal deaths. The collation of these cases into the tool highlighted emerging trends. In the case of Suhul Hospital, this was severe anaemia leading to cardiac or multi-organ failure, which is not among the usual leading causes for maternal mortality [21]. R-MDSR’s identification of antenatal anaemia as a major concern for women attending hospital was suspected to indicate broader issues for pregnant women in the surrounding areas. This was followed-up by community outreach teams who explored further possible underlying causes, including conflict-related food insecurity [22], malnutrition and disruption to antenatal services (including access to iron supplementation, malaria and helminth prevention). These hypothesises were used to further inform plans for medical humanitarian activities outside the facility and beyond the initial response phase.

During the pilot testing, the R-MDSR tool was evaluated, altered and adapted in real-time to meet gaps identified by the teams. The quality improvement (action) points identified through R-MDSR tool were advocated for by the country team. Simultaneously, MSF staff working in the hospital continued updating the tool as further details emerged and as additional maternal deaths were reported. This dynamic process played a crucial role in sustaining relevant and focused action plans alongside implementation of medical humanitarian activities.

## Reflections and limitations

R-MDSR was developed and piloted in reaction to an ongoing humanitarian emergency. Consultation and planning happened alongside implementation, which was critical for ownership and to enable usability by end-users and decision makers. While this allowed for tweaking of the tool in real time, it limited the ability for more detailed research into tool development and formal user feedback. Nonetheless, the institutional and context-specific knowledge of local actors was vital in understanding underlying factors and implementing a suitable response. Studies and reviews on MPDSR in humanitarian settings, published since the R-MDSR tool was developed and piloted, also demonstrate and call for customized approaches to implementing MPDSR which respond to the needs of the health workers and aid workers on the front lines [23, 24].

The R-MDSR tool has continued to be used past the initial pilot phase, going on to identify several other key action points. These included focussing on the hospital entry-point and emergency department to reduce treatment delays, training packages for health workers and emergency equipment preparedness for birth complications. At the time of writing this Comment, the tool continues to be utilised by MSF-OCA at Suhul Hospital and a second location in Tigray. During MSF-OCA’s response to the civil war in Sudan, R-MDSR has been implemented in the eastern area and in Darfur. In each setting, key factors for maternal deaths were identified, tailoring the humanitarian response [25].

The use of the R-MDSR tool has also been effective as an advocacy tool, slotting maternal health into organisational strategic discussions, in front of key decision makers and provided evidence to generate support for context-specific responses. The maternal mortality reviews and their findings offered greater visibility to maternal health within a crowded medical-humanitarian space and amongst competing priorities. The initial R-MDSR findings, and the sustained impact of the resulting interventions, provided evidence to maintain ongoing support for activities in the maternity unit, and specialised clinical area, which has continued to admit women for over a year since the pilot phase.

The austere environment during and after the conflict in Tigray was limiting in several ways. In addition to the scarcity of medical supplies and reduced human resources, the internet coverage was unpredictable, often absent, precluding access to online resources. This pilot was at one facility and small in numbers. There were numerous gaps in the data, making it incomplete and limited in value. While this was anticipated given the conflict and humanitarian crisis, drawing firm conclusions from the data retrieved from the R-MDSR tool would need to be made with caution.

## Conclusion

This Comment reflects on the development and pilot testing of a R-MDSR tool designed to assist frontline health workings, including those supported by the MSF-OCA team, to identity and implement targeted responses aimed at reducing maternal deaths, during a humanitarian crisis. The information collected through the tool enabled rapid reviews of cases to identify underlying factors related to maternal mortality and informed the response and advocacy for maternal health among many other competing humanitarian needs.

Further implementation of community and facility MPDSR-related processes in humanitarian crises is necessary to improve the maternity response to affected populations. With further research, R-MDSR, may be able to meet this need without overburdening stretched medical teams.

## Supplementary Information

Additional file 1.

Additional file 2.

## Data Availability

The datasets generated and/or analysed during the current study are not publicly available due to patient confidentiality and security but are available from the corresponding author on reasonable request pending MSF and Ethiopia MOH approval.

## Abbreviations

MDSR: Maternal Death Surveillance and Response
MOH: Ministry of Health
MPDSR: Maternal and Perinatal Death Surveillance and Response
MSF: Médecins Sans Frontières
OCA: Operational Centre of Amsterdam
R-MDSR: Rapid Maternal Death Surveillance and Response
UNFPA: United Nations Populations Fund
WHO: World Health Organization
