# Supplementary material for: Lessons learned from applying a “Rapid Maternal Death Surveillance and Response” tool in conflict-affected Tigray, Ethiopia
Source: BMC Pregnancy Childbirth. 2025 Oct 6;25(Suppl 1):1017. doi: 10.1186/s12884-025-08182-y (PMC12498451; doi:10.1186/s12884-025-08182-y)
Supplement: Supplementary file 1 — Additional file 1. [file 12884_2025_8182_MOESM1_ESM.docx]

**Additional File 1:**

**R-MDSR Structure**

The structure of the R-MDSR tool was split into three parts: A, B and C. These corresponded to the record of the death (or near miss), the review and analysis to identify underlying causes, and the action points, respectively.

The key demographic and clinical aspects of the woman and her death were recorded in part A. Enabling the team to be able to identify patterns in geographic spread, timeline and causes of deaths.

Part B was designed to support identification of root causes and plan for action points. While the ideal scenario involved adhering to the traditional MPDSR approach of regular committee meetings with all stakeholders, a flexible approach was adopted, accounting for the constraints of the emergency setting, allowing the team to opt for either case-by-case discussions or smaller sub-group interactions if deemed more feasible. To guide the reviewers towards identification of underlying factors and action points the tool used a similar formula seen in After Action Reviews (AAR).

^[[1]](#endnote-2)^ This is a simple structured approach to learning from an event usually through a facilitated discussion. The AAR objective is to identify gaps, lessons and future action points through asking the questions; What happened? What should have happened? Why was there a difference?^[[2]](#endnote-3)^ This leads the team to the final section, Part C, recording what could be learnt and what actions would reduce the risk of the same situation happening again. The findings of A, B and C are transcribed across into an excel spreadsheet, with patient details anonymised. Once the R-MDSR is in operation the spreadsheet should be reviewed on a regular basis for identification of concerns, patterns and action points.

1. World Health Organization. Highlighting the benefits of an after-action review. [Internet]. WHO; 2023 [cited 2023 Dec 4]. Available from: https://www.who.int/europe/activities/highlighting-the-benefits-of-an-after-action-review [↑](#endnote-ref-2)
2. National Health Service (NHS) England. After Action Review. [Internet]. NHSE; 2023 [cited 2023 Dec 4].Available from: https://www.england.nhs.uk/wp-content/uploads/2021/12/qsir-after-action-review.pdf [↑](#endnote-ref-3)
