# Supplementary material for: Lessons learned from applying a “Rapid Maternal Death Surveillance and Response” tool in conflict-affected Tigray, Ethiopia
Source: BMC Pregnancy Childbirth. 2025 Oct 6;25(Suppl 1):1017. doi: 10.1186/s12884-025-08182-y (PMC12498451; doi:10.1186/s12884-025-08182-y)
Supplement: Supplementary file 2 — Additional file 2. [file 12884_2025_8182_MOESM2_ESM.docx]

**What is R-MDSR?**

Maternal Death Surveillance and Response (MDSR) is a system for recognising, recording and learning from deaths during pregnancy and birth. The Rapid-Maternal Death Surveillance and Response (R-MDSR) is a tool for reviewing maternal deaths in settings which lack the capacity or resources for a full maternal mortality review process, for example in emergency responses, in contexts overwhelmed by clinical activity or during assessments. It can also be used for near miss cases. R-MDSR can be used for community or facility deaths.

**Maternal deaths have far reaching consequences. They often signal other underlying problems within a society.**

**ALL MATERNAL DEATHS in all situations should be reported and reviewed to the *full extent possible*** – an incomplete review is acceptable given the context.

This will support in guiding where to focus life-saving activities.

**Common Definitions**:

| **Maternal Death** | The death of a woman while pregnant or within 42 days of the end of pregnancy. |
| --- | --- |
| **Near Miss** | Survival of a life-threatening condition arising from complications related to pregnancy or childbirth. |
| **Community maternal death** | A death which occurs outside a health facility (e.g. at home) |
| **Facility maternal death** | A death which occurs in the health facility (e.g. community health post or hospital) |

**Active maternal death case finding and notification** – maternal deaths can often go unnoticed or uncounted. It is important to ensure ongoing sensitization with colleagues and community so that everyone knows how and who to inform if a woman has died during or within 6 weeks of ending pregnancy.

Teams should **actively seek-out and follow-up on suspicions or rumours of a death either in the community** (e.g. incorporating into routine disease surveillance activities) **or in a facility** (e.g. asking about the mother during newborn consultations and checking on non-maternity departments such as ER).

**How to use R-MDSR**

The purpose of R-MDSR is to ensure that, as a minimum, maternal deaths in the community and at facilities are recognised, recorded and lessons learnt. When the situation allows the team should transition to the standard version of MDSR (see facility and community tools on SharePoint available [**here**](https://msfintl.sharepoint.com/sites/OCA-dept-PHD/SitePages/Maternal-Mortality.aspx)**)**.

**No name / no blame** – teams may be reluctant to notify or review maternal deaths if this is thought to reflect badly on them or result in disciplinary actions. It should be clear that R-MDSR is a way of improving care to a population. MDSR is a learning event, not a critique. No names of staff should be used in the review of a maternal death. Names of the deceased must be treated with confidentiality.

**Approaches to R-MDSR:**

1. Complete as much as possible of the R-MDSR form as soon as possible after each death (either as an individual or team exercise).

*or*

1. Complete as much as possible of Parts A & B of R-MDSR as soon as possible, then have a meeting each month to discuss all deaths and complete Part C as a team exercise.

**Recording R-MDSR Findings:**

To maintain memory of learning events and identify trends in maternal deaths (to guide future operations) it is vital the details are recorded. Give completed R-MDSR forms to the focal person (usually MWAM or MTL) so the details can be transcribed across to R-MDSR spreadsheet (see embedded version below or request from SRH advisors).


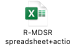


**Record actions that should be taken to reduce future maternal deaths, even if they cannot be completed at the time of recording** (e.g. due to high work load or emergency context) – they will still be useful for the future and for understanding gaps in the service. Where actions are implemented these should be recorded on the final page of the spreadsheet (e.g. success of implementation).

**Make actions specific and simple** – Try to keep actions realistic, the more specific an action is the more likely it is to be achieved, e.g. ‘Provide an ambulance to the hospital’ is more specific than ‘Improve transport’, it is also more achievable.

**Involve coordination and advisors** – Each maternal death is a significant event. Where concerns are identified which need urgent attention these should be raised with coordination. Should it be necessary, further support can be requested from the SRH advisors or other appropriate advisors.

**R-MDSR findings should be incorporated into the weekly sitrep or monthly medical report** **(MMR)**. This should not include any patient or staff identifiable details.

*If reviewing a Near Miss case, use same questions and add “nearly”. For example, When did they* ***nearly*** *die? Where did they* ***nearly*** *die? Etc*.

| **Part A** | | | | | | | | |
| --- | --- | --- | --- | --- | --- | --- | --- | --- |
| **Date of Review** |  | | | | | **Case Number** |  | |
| **Who is your information source?** | | - Doctor - Midwife - Traditional Birth Attendant (TBA) - Other health worker - Relative - Other (specify) | | | | | | |
| **Who died? (first three letters of name, if known)** | |  | | | | Age: | | |
| **When did they die?** | | Date of Death: | - Before Birth - During Birth - After Birth | | | Estimated pregnancy gestation when death occurred:  or, time since birth (if a postnatal death): | | |
| **Where did they die?** | | Home | | | Travelling | Facility | | Exact location name: |
| **What was the suspected cause of death?**  e.g. post-partum haemorrhage, eclampsia, sepsis | | | |  | | | | |

| **Ask if there are rumours or known cases of other maternal deaths?** | Yes / No | **If YES, start a new R-MDSR form**  (even if minimal information) |
| --- | --- | --- |

Complete Parts B & C to the *full extent possible* for each death

| **Part B** | | **Case Number:** |  |
| --- | --- | --- | --- |
| **What happened?**  Briefly describe, using words/information of interviewee  e.g. delays, staff issue, complication in management, equipment issue… | |  | |
| **In an “ideal situation” what should have happened?**  According to interviewee & external opinion (e.g. MTL/MWAM) | |  | |
| **Why was there a difference?**  According to interviewee & external opinion | |  | |

| **Part C** | | | **Case Number:** |
| --- | --- | --- | --- |
| **What can be learnt for the future?**  According to interviewee &/or external opinion |  |  |  |
| **What actions would help reduce the risk of this happening again?**  What can MSF do now or in the future to prevent maternal deaths (include MTL/MWAM in deciding these) |  |  |  |

This form should be given to the R-MDSR focal person (usually MTL or MWAM) and transcribed across to the R-MDSR spreadsheet.

*Even if not complete or immediately actionable: It is better to record something, rather than recording nothing at all.*

Rapid-MDSR V1. 2022. MSF OCA
